# A Health Information Technology Protocol to Enhance Colorectal Cancer Screening

**DOI:** 10.2196/55202

**Published:** 2024-04-19

**Authors:** Adam Baus, Dannell D Boatman, Andrea Calkins, Cecil Pollard, Mary Ellen Conn, Sujha Subramanian, Stephenie Kennedy-Rea

**Affiliations:** 1 Department of Social and Behavioral Sciences School of Public Health West Virginia University Morgantown, WV United States; 2 Cancer Prevention and Control West Virginia University Cancer Institute Morgantown, WV United States; 3 Implenomics Dover, DE United States

**Keywords:** electronic health record, EHR, colorectal cancer screening, health information technology, cancer, colorectal cancer

## Abstract

This study addresses barriers to electronic health records–based colorectal cancer screening and follow-up in primary care through the development and implementation of a health information technology protocol.

## Introduction

Cancer is a pressing global public health problem and the second leading cause of death in the United States, accounting for an estimated 1670 deaths daily [1]. Colorectal cancer (CRC) is the third most commonly diagnosed cancer, the second leading cause of cancer death worldwide [2], and the third most common cause of cancer-related deaths in the United States [3]. More effective use of health information technology (HIT), including electronic health records (EHRs), can aid in improving CRC screening and care [4]. Studies from as early as the 1990s have shown that EHRs and associated clinical decision support tools have promise in helping with patient care and population health needs [5]. However, barriers like clinician readiness [6] and clinical workflow integration [7] hinder EHRs’ full benefits. This study aims to address barriers to EHR-based CRC screening and follow-up through the development and implementation of a universally applicable EHR protocol tailored to identify and overcome practice workflow and EHR challenges.

## Methods

### Overview

This study used a mixed methods approach, involving quantitative and qualitative data collection techniques, conducted across 3 diverse health systems in West Virginia to develop and implement an EHR protocol for CRC screening and follow-up. These health systems were purposefully chosen to encompass diverse sizes, organizational structures, geographic locations, patient demographics, and EHR preferences, thereby supporting the generalizability of the study’s findings. These included a free and charitable clinic, a larger, urban, federally qualified health center, and a smaller, rural, federally qualified health center. Key stakeholders, including health care administrators, clinicians, and information technology personnel, were identified as potential participants. This study was conducted from April 2021 through April 2022. Implementation mapping methodology guided the assessment of current CRC screening practices and the development, implementation, and evaluation of the EHR protocol. Data collection tools were pilot tested in Health System A to assess their reliability, validity, and feasibility, then refined prior to full implementation in Health Systems B and C to ensure quality and effectiveness in data collection. Evaluation of the protocol’s acceptability, appropriateness, and feasibility was conducted using the Acceptability of Intervention Measure (AIM), Intervention Appropriateness Measure (IAM), and Feasibility of Intervention Measure (FIM). Technical issues during the study were resolved collaboratively by the research team and technical staff through troubleshooting, protocol adjustments, and ongoing support.

### Ethical Considerations

This study received ethics approval from the West Virginia University Institutional Review Board (protocol number 2107363377).

## Results

The development of the EHR protocol involved a collaborative process between the research team and key stakeholders from participating health systems. Initial assessments revealed common challenges in CRC screening and follow-up across the diverse settings, including issues related to data quality, workflow inefficiencies, and underutilization of EHR functionalities. Based on these findings, a draft protocol was formulated, emphasizing strategies to enhance EHR data quality and optimization specifically tailored to address the identified barriers. The protocol comprised three key components: (1) *Quality Improvement Activities*, guiding clinic staff through a Plan-Do-Study-Act cycle to identify and mitigate data entry errors; (2) *EHR Optimization Factors*, highlighting specific EHR features supporting CRC screening and follow-up when effectively used; and (3) *Health Information Technology Assessment*, facilitating structured discussions on EHR use roles, office workflows, knowledge, skills, abilities, challenges, and improvement opportunities.

The developed protocol was implemented in Health Systems B and C following its refinement based on feedback from the development site (Health System A). Implementation involved training sessions for clinic staff on protocol utilization and ongoing support from the research team. Eight staff members from the participating health systems completed the AIM, IAM, and FIM assessments, providing valuable insights into their perceptions of the protocol. The mean scores from AIM (mean 16.00, SD 4.24), IAM (mean 15.80, SD 4.54), and FIM (mean 16.80, SD 4.66) indicate favorable perceptions of protocol feasibility, acceptability, and appropriateness. Qualitative feedback from participants further supported the positive reception of the protocol, with respondents expressing satisfaction with its efficacy and intentions to integrate it into their clinical practices. All respondents indicated that they would use or would consider using the protocol within their clinics again. Open-ended responses included “very pleased with the protocol and leveraging EHR/staff/outreach” and “plan to now identify and track to completion of CRC testing.”

## Discussion

The results demonstrate the successful development and initial implementation of an EHR protocol aimed at enhancing CRC screening in primary care settings. The protocol’s favorable reception by clinic staff, as indicated by high scores on acceptability, appropriateness, and feasibility measures, suggests its potential effectiveness in addressing identified barriers. The diverse representation of health systems and EHR platforms involved in the study enhances the generalizability of findings. Limitations include the small sample size and the focus on a specific geographic region. Future research will assess the protocol’s performance across additional EHR systems and health care settings for enhanced scalability and further evaluate the protocol’s impact on CRC screening outcomes.

## Abbreviations

AIM: Acceptability of Intervention Measure
CRC: colorectal cancer
EHR: electronic health record
FIM: Feasibility of Intervention Measure
HIT: health information technology
IAM: Intervention Appropriateness Measure
